# Planning for change: Transformation labs for an alternative food system in Cape Town, South Africa

**DOI:** 10.1186/s42854-020-00016-8

**Published:** 2020-11-17

**Authors:** Laura Pereira, Scott Drimie, Olive Zgambo, Reinette Biggs

**Affiliations:** 1 Centre for Complex Systems in Transition, Stellenbosch University, Stellenbosch, South Africa; 2 Centre for Food Policy, City University of London, London, UK; 3 Stockholm Resilience Centre, Stockholm University, Stockholm, Sweden; 4 Southern Africa Food Lab, Stellenbosch University, Stellenbosch, South Africa

**Keywords:** Co-production, Food systems, Governance, Participatory approaches, South Africa, Sustainability, Sustainability transformations, T-labs, Transformative space

## Abstract

There has been a call for more participatory processes to feed into urban planning for more resilient food systems. This paper describes a process of knowledge co-production for transforming towards an alternative food system in Cape Town, South Africa. A ‘transformative space’ was created though a T-Lab process involving change-agents advocating for an alternative food system, and was designed to discuss challenges in the local food system from a range of perspectives, in order to co-develop potentially transformative innovations that could feed into government planning. In this paper, we describe and reflect on the T-lab in order to consider whether its design was able to meet its objective: to initiate an experimental phase of coalition-building by diverse actors that could feed into the provincial government’s strategic focus on food and nutrition security. Our findings indicate that T-labs have the potential to be important mechanisms for initiating and sustaining transformative change. They can be complementary to urban planning processes seeking to transform complex social-ecological systems onto more sustainable development pathways. However, as with all experimental co-production processes, there is significant learning and refinement that is necessary to ensure the process can reach its full potential. A key challenge we encountered was how to foster diversity and difference in opinions in the context of significant historical legacies of inequality, whilst simultaneously acting for ‘the common good’ and seeking ways to scale impact across different contexts. The paper concludes with deliberations on the nature of planning and navigating towards systemic transformative change.

## Introduction

Cities are hot spots of change in the Anthropocene and are critical areas for engaging with sustainability transformations (McPhearson et al. 2016). In the global South, where countries are undergoing rapid urbanization, it is critical to engage with both the challenges and solutions for living sustainably and equitably on an urban planet (Elmqvist et al. 2018; Nagendra et al. 2018). Novel governance and planning tools are starting to emerge from these places where sustainability transformations are ‘life-and-death’ matters of the present, not merely a specter of a potential future. Cities like Cape Town, South Africa, are places facing a legacy of unequal access to resources like food, housing, water and sanitation. At the same time, the city also has to face the challenges brought on by global environmental change, such as the drought that almost brought the city to its knees during 2016–2018. Learning from places that are grappling with these complex challenges can provide broader insights for encouraging decisionmaking processes that are able to consider the complex challenges and uncertainties of navigating the Anthropocene.

It is increasingly being recognized by the scientific and practitioner communities that shifting onto a more sustainable trajectory for people and the planet requires transformative change, i.e. fundamental restructuring of current social, political, economic, and technological systems to make sustainability the norm and not the exception (O’Brien 2012; Olsson et al. 2017; Díaz et al. 2019). One of the systems that lies at the heart of the sustainability challenge, which is in desperate need of transformative change, is the food system (Nyström et al. 2019; Pereira et al. 2019b; Willett et al. 2019). Historically, food-related challenges were largely related to production and focused on rural areas (Ericksen et al. 2010; Ingram 2011). However, the increasingly important role of cities, and in particular urban food policy, in addressing the contemporary challenge of ensuring that all citizens have access to sustainable, healthy and culturally appropriate food has been recognized (Haysom 2015; Battersby 2017a; IPES-Food 2017). Thus, there is a clear link between improved urban planning to foster transformative change in the food system, and, similarly, a need to focus on how addressing the complex challenges of food system transformation can help inform better urban planning for sustainability that considers complexity and diversity. In this paper, we are primarily concerned with the latter-how processes developed for enabling transformative change in the food system can inform urban planning processes and help cultivate novel ways of making decisions in urban settings.

The term ‘transformative spaces’ has been proposed to describe processes of coproduction that aim to galvanize transformative change towards sustainability (Pereira et al. 2018a). One such example of a transformative space is a Transformation Lab or T-Lab (Ely et al. 2020). T-Labs are specifically designed to guide transformations in *social-ecological systems* towards sustainability, by supporting changes in the conditions that made these systems unsustainable in the first instance. These processes include a set of stakeholders who may have different roles and perspectives, but who have a common interest in solving “the problem” (see The Pathways Network 2018 for more on T-lab design). T-labs are still a “new and experimental concept” and until recently, have mostly been used as a tool towards sustainable transitions in Western contexts, although a recent research agenda has showcased the incredible learning that can come from initiating these spaces in other places (Ely et al. 2020; Pereira et al. 2020a). Despite these recent advances, there remains a research gap in better understanding their application to the unique social, political, economic and ecological dynamics of the global South where many of the complex challenges of the Anthropocene are already playing out, but also where many of the solutions are being initiated (Pereira et al. 2020a). To the best of our knowledge, the T-labs held in South Africa were the first to have been undertaken in the global South, although there have now been subsequent T-labs as part of The Pathways Network (2018), for example in Mexico (Charli-Joseph et al. 2018) and Argentina (van Zwanenberg et al. 2018) as well as China and Kenya (The Pathways Network 2018).

In this paper, we explore T-labs as participatory processes bringing together change-agents to generate outcomes with which policy makers and planners can engage and that they can use to inform and shape systemic interventions. We focus on the food system as an entry point for bringing diverse stakeholders together for a deep engagement about systemic change. The overarching research question was to explore how the design of a T-lab can shape its potential to serve as an intervention for food system transformation in the urban context of Cape Town. The T-lab approach comes with an underlying assumption that systemic transformations result from bottom-up, marginal processes interacting with larger-scale changes to shift the dominant regime of how things are done. This is a theory of change explicit in most social-ecological systems transformations literature (Westley et al. 2013; Moore et al. 2014; Olsson et al. 2014; Pereira et al. 2020b) as well as the literature on socio-technical system transitions (Geels 2002, 2011; Geels and Schot 2007; Grin et al. 2010), as elaborated in section 3.

A key challenge in studying the efficacy of interventions is the difficulty in tracking transformative change. In this study, we restricted our focus to exploring whether the design of a T-lab was able to meet the objective of creating a transformative space that could initiate an experimentation phase of coalition-building by navigating diverse experiences. It was envisaged that the process would build new relationships and strengthen the networks within the alternative food system, and thus serve as a platform for developing solutions to the challenges faced by the participants that could then feed into strategic planning on food and nutrition security. A particular interest was to see how the outcomes from the T-lab could feed into an ongoing provincial planning process that sought to incorporate novel methods for including diverse stakeholder voices in its policy development process (See Adelle et al. 2019).

We start by describing the sustainability challenge of transforming the food system in the Western Cape province, South Africa and how this is part of a broader need to develop ways to enable positive transformative change. We then go on to describe the intervention of two T-labs in South Africa, held in 2016 and 2017, to engage with food system transformation, as new methods for collectively coming to terms with how to act to change the status quo. The discussion reflects on the learnings from running the T-labs and how the design was able to meet its objectives in some instances, but also where the process fell short. We also discuss how the development of a provincial Food Charter was influenced by the T-lab processes, and influenced the operationalisation of a government-led Food Declaration for the province. We conclude with reflections on the need for more such co-produced processes to intertwine with government-led approaches in order to create enabling environments for transformative change towards sustainability. In particular, we emphasize how a wider set of cases from around the world, particularly those from under-researched areas in the global South, are critical to improving our understanding of how to initiate and follow through with participatory processes aimed at enabling longterm sustainability transformations.

### The food system as a wicked challenge in Cape Town, South Africa

Currently, the global food system is impacted by many ecologically unsustainable and socially unjust dynamics and food production practices that lead to undesirable consequences for people and the environment (Steffen et al. 2015; Gordon et al. 2017). As a complex social-ecological system (SES) with diverse actors and logistics that “spread across time and space” and that exhibits behaviour typical of complex adaptive systems, the food system is influenced by social, political, economic and environmental factors (Ingram 2011; Pereira and Drimie 2016; May 2017). These include pressures from climate change, increased demand due to rapid population growth, and the increased reliance on trade to fulfil food and agriculture requirements globally (Oliver et al. 2018; Willett et al. 2019).

In South Africa, these pressures manifest from the national through to local levels. South Africa is prone to local food insecurity and hunger despite being food secure at the national level and a large exporter of grains, livestock, stone fruit and wine (Zgambo 2018). The country is plagued by social, economic and ecologically unsustainable food practices that render 23–30% of the population with inadequate access to food or risk of hunger (Shisana et al. 2014; Ledger 2016; Mbhenyane 2016). Hunger and malnutrition persist due to “complex and interrelated [...] environmental, health, economic, socio-political and agro-food issues”, including increasing unemployment, food price volatility, HIV and AIDS, drought conditions, a decrease in government support for agriculture, and persistent high levels of urban and rural poverty (Drimie and McLachlan 2013: 218). In addition, a few large corporations currently dominate the agricultural sector and the production, distribution, processing and marketing of food and its subsequent products (Greenberg 2017; May 2017). The effects of this market domination are intensive in urban areas where most of the population do not have access to land on which to grow their food, nor access to healthy food options such as fresh fruit and vegetables, lean meats and fish (Faber et al. 2011; Temple and Steyn 2011). Instead, they resort to cheaper, more affordable energy dense diets of refined cereals, sugars and salt (Igumbor et al. 2012; Hofman and Tollman 2013; Pereira 2014; Mbhenyane 2016). Although South Africa has an entrenched constitutional right to food, policy has been slow in moving towards fulfilling this right (Drimie 2015; Mclaren et al. 2015; Moyo 2016; Pereira and Drimie 2016).

The Western Cape is one of the nine provinces of South Africa and is home to over 5.82 million people on 129,370 km^2^ of land with its capital the city of Cape Town. Compared to other provinces, it has a relatively prosperous and well-established food system, nevertheless, it is not meeting food security needs and is vulnerable to environmental pressures such as drought. Many households do not have access to adequate food, and even more children are at risk of malnourishment (Frayne et al. 2009; Battersby 2011; Shisana et al. 2014; Mbhenyane 2016). Negative impacts caused by climatic changes such as increased temperatures, decreased winter rainfall, longer dry spells and more frequent droughts are some of the contributing factors exacerbating the Western Cape’s vulnerability to food insecurity (ACDI 2016). The province is also subject to some of the trends that shape South Africa’s agrarian sector, including white commercial farmer dominance over many black subsistence farmers, large corporate company control over available or accessible food, and increased food waste (Pereira and Drimie 2016). Without any clear or shared understanding of a pathway toward a more sustainable configuration, this situation could rapidly worsen to the detriment of the most vulnerable in society.

Cape Town is the biggest city in the province and home to over 3.7 million people. Here, urban agriculture has become a food security strategy (Frayne et al. 2009). The Philippi Horticultural Area (PHA), an agricultural zone in the heart of urban Cape Town, is responsible for about 100,000 t of Cape Towns’ annual fresh produce, estimated to be 80% of the city’s vegetable needs (Battersby-Lennard and Haysom 2012). However, despite this prevalence of fresh produce, most of the urban poor (many of them living in the informal settlements near to the PHA) increasingly rely on supermarkets-many of which sell more highly processed, energy-dense food that is low in nutrition and devoid of dietary diversity (Battersby and Peyton 2014, 2016; Peyton et al. 2015). The diets of most low income households therefore comprise energy-dense foods including refined cereals, sugar and fat, with little to no nutrient-dense foods like lean meats, fish, vegetables and fruit (Temple and Steyn 2009; Mbhenyane 2016; Faber and Drimie 2016). The safety of these foods are also compromised by challenges that street food vendors face, including lack of access to safe water to prepare the food, refrigeration, and/or basic food safety training (Even-Zahav 2016; Boatemaa et al. 2019). Battersby (2017a, 2017b) argues that the local government is playing a profound role in reshaping the food system through non-food related planning and policy decisions designed to achieve urban development objectives. She uses Cape Town as a case study of a rapidly changing food system to show how the urban planning agenda-for example plans to develop the PHA away from agricultural production-is inadvertently generating a food system that undermines food and nutrition security and suggests new opportunities for more inclusive urban food systems planning are essential (Battersby 2017b).

In 2016, the provincial government through the Department of the Premier led the development of the Western Cape Household Nutrition and Food Security Strategy, also known as Nourish to Flourish (Western Cape Government 2016). This initiative adopted a system wide approach that identified key opportunities to protect the availability and stability of food supply as well as improving access and utilisation for all households in the province, particularly those that are the most vulnerable to hunger. As researchers who had been involved in this process, particularly to help convene consultations around the strategy, it provided a key opportunity to engage with more participatory processes at city-level that could feed into the provincial implementation of this strategy. The ongoing interest in an urban food agenda, coupled with the researchers’ existing links to provincial government and other alternative food actors, presented an opportunity to explore how the opportunity context created by a larger-scale planning process could be informed by T-labs-as processes for actively enabling transformative change led from below.

### Sustainability transformations and T-labs

A departure from current food system dynamics towards more sustainable urban food systems requires radical transformation (Bennett et al. 2016; Pereira et al. 2019a; Willett et al. 2019). Such a process of transformation would entail deep systemic changes to existing ecological, social, economic or political conditions (Olsson and Galaz 2012; Folke et al. 2014; Ely and Marin 2016). A recent analysis of transformations perspectives highlights that there is a need to combine structural, systemic and enabling approaches to sustainability transformations for more effective impact (Scoones et al. 2020). Conceptual links between different approaches have already been developed in the study of sustainability transformations (Pereira et al. 2015). A diverse literature from both social-ecological systems (SES) thinking as well as socio-technical systems argues that transformation processes are caused by the dynamic interaction across different levels of a system (Geels and Schot 2007; Olsson et al. 2014). Transformations can occur when niche-innovations at the local level build up internal momentum, whilst contextual changes create pressure on the dominant system (regime) to shift (Olsson and Galaz 2011; Olsson et al. 2014). Destabilisation of the current regime can create a window of opportunity for niche-innovations to form new regimes (Pereira et al. 2020b). When changes at multiple levels reinforce each other, systemic transformation can occur (Geels and Schot 2007; Avelino et al. 2017).

Drawing on these theoretical approaches and putting them into practice through research co-production processes (or ‘enabling transformations’) has been termed creating ‘transformative spaces’ (Pereira et al. 2018b; Scoones et al. 2020). The concept of ‘transformative spaces’ is defined as ‘“safe-enough” collaborative environments where actors invested in transformation can experiment with new mental models, ideas, and practices that can help shift social-ecological systems onto alternative development pathways [...] they allow and enable dialogue, reflection, and reflexive learning, while reframing issues in ways that allow solutions to be co-created and co-realized’ (Pereira et al. 2018b: 2). Transformative, “safe” or “safe enough” spaces are vital in transformation processes (Pereira et al. 2018b). Safe spaces are about experimentation, and creating opportunities for newness to emerge at small scales and then spread across larger scales (Ely and Marin 2016; Pereira et al. 2020a). The experiments in safe spaces look different in different contexts, but they have a common goal towards developing disruptive innovations that can create new SES pathways (Feola and Butt 2017). Innovations can be either social or technical, but both often work in conjunction to address a challenge or need in society or bring about positive futures. T-labs, as “safe enough” spaces foster the development of niche activities, thus facilitating SES transformations towards more sustainable trajectories (Zgambo 2018). They should shield, nurture and empower emerging innovations from pressures emanating from the dominant system and provide a holding or temporary protective space for innovative ideas or activities to develop (Smith and Raven 2012).

Transformation labs (T-labs) draw on and further develop the concept of social innovation labs to incorporate social-ecological dynamics (Ely and Marin 2016; Charli-Joseph et al. 2018; van Zwanenberg et al. 2018). The contribution of these labs to transformation, include experimental methods, a transdisciplinary mode of research, scalability and transferability of results, as well as scientific and societal learning and reflexivity (Schäpke et al. 2018). Other similar examples include living labs (Bergvall-Kåreborn and Stahlbrost 2009; Bergvall-Kåreborn et al. 2009; von Wirth et al. 2019), real-world labs (Schäpke et al. 2015, 2018), urban living labs (Voytenko et al. 2016; Bulkeley et al. 2016; Naumann et al. 2018) and urban transition labs (Nevens et al. 2013) as well as systems oriented processes in global South contexts (Bosch et al. 2013; Nguyen and Bosch 2013; Banson et al. 2016). Recent development from the transgressive learning space have also provided valuable insights on co-production processes for transformative change (Lotz-Sisitka et al. 2015; Mukute et al. 2018). Unfortunately, it is not possible to go into a thorough review of these lab processes here, but they contribute important knowledge to the T-lab process and have been important for the development of the concept of ‘transformative spaces’ (See Sengers et al. 2016; Pereira et al. 2020a).

T-labs are therefore not revolutionary new concepts, but draw from a variety of existing lab-based methodologies. Based on (Zgambo et al. (2018), a T-lab is a space for:

- facilitated, collective learning about the nature of a problem or challenge;
- learning about different kinds of possible solutions, or pathways of possible change;
- helping to create a collective sense of the need for change – within and beyond the stakeholders directly involved;
- identifying strategies for affecting change; and
- identifying which actors have transformative power.

T-labs are intervention processes that require thorough planning, but are still flexible enough to allow emergence and the unexpected to occur. Ideally, the form a T-lab takes is dependent on the local context and the people involved. Drawing on the Western Cape context (see Zgambo 2018), we propose that the following are some of the conditions under which a T-lab may be an effective intervention:

1. There is a complex SES challenge to address
2. A diverse group of participants with potential for transformative agency exists
3. There is an identifiable action-oriented outcome as the end goal of the process
4. There is a convenor who is strongly motivated to ensure longer-term engagement in the process
5. There is tension in the regime, or noticeable shifts in the culture or economic or political scene that can serve as potential windows of opportunity for T-lab innovations to take effect.

T-labs offer a methodological approach for working with the emergence of bottom-up and collaborative planning initiatives targeting urban sustainability and transformation. As researchers are finding themselves at the intersection of action and analysis, where they navigate the fine line between actively intervening in processes to enable change, whilst also being able to provide a critical analysis of what types of changes are occurring, some researchers are finding themselves as ‘transformative space-makers’ (Marshall et al. 2018). T-labs are an example where research has opened up a space for productive collaboration and interaction between diverse stakeholders with the intention that there may be actionable outcomes with which policy and other decisionmaking actors can engage. However, as highlighted by Schäpke et al. (2017), empowerment, social learning and social capital development are important aspects in facilitating transformations, but have been relatively under-developed in research. In the case of the Western Cape T-lab, we focus more specifically on the development of social capital and in particular the need for networking and coalition-building amongst marginal actors (Ziervogel et al. 2016; Termeer et al. 2018). We posit that an emphasis on social capital development through networking and coalition building is central to enabling transformative change, building on similar work on real-labs in Germany (Schäpke et al. 2015) and in niche learning in South Africa (Metelerkamp et al. 2020).

The overarching research question was to see whether a T-lab can serve as an intervention for food system transformation in the South African context. Transdisciplinary sustainability research projects can lead to three basic outcomes: outputs in the form of usable products, impacts in the form of enhanced capacities and network effects, such as new relationships, trust or accountability (Wiek et al. 2014). As such, we sought to determine whether the design was able to meet its objective to create a transformative space that could initiate an experimentation phase of coalition-building by navigating diverse experiences in order to result in both an actionable output as well as new relationships being formed. By focusing on two of the five thematic propositions put forward by Zgambo et al. (2018)- looking at diversity (2) and action-oriented outcomes (3)- it is also possible to see how these reflections on methods and outcomes connect to the more theoretical descriptions of T-lab processes as transdisciplinary sustainability research.

## Methods

### Designing the T-labs

As mentioned above, T-labs are a relatively new methodological concept. The design of the labs was therefore highly experimental and the researcher-facilitators learned a lot about T-lab process design over the 2 years. There is no strict methodological process to a T-labs, but a useful starting point is the Social Innovation Lab Guide (Westley and Laban 2012) that sets out how a participatory process focused on a complex challenge can be designed not only to imagine high potential interventions, but also to gain system insight, redefine problems, and identify opportunities in the broader context with the potential to tip systems in positive directions. The aim of this study was to experiment with T-labs as a way to connect key niche innovators in order to start experimenting with how to build a viable alternative food system in Cape Town. The desired outcome was to connect these actors and generate action-oriented outcomes that could feed into more participatory planning processes. The T-labs were therefore designed to create bridges, by linking chefs to producers, restaurateurs to informal traders, and academics to actual work on the ground. This connection and process was an opportunity to re-imagine the ways in which food is produced, processed and consumed and potentially to become more embedded, and strategically aligned to influence the dominant food system.

Following a consultation workshop with researchers interested in the Cape Town food system in December 2015 at the Stellenbosch Institute for Advanced Studies, it was decided to proceed with participatory action research in this area. Two T-lab processes were conducted in November 2016 and in July 2017 to serve as a platform for dialogue to harness the potential for food system transformation in the broader Cape Town area (see Tables 1 and 2). Ethics approval for these workshops was granted by Stellenbosch University Departmental Ethics Screening Committe, reference number SU-HSD-004283 and all participant ssigned consent forms agreeing to the use of their images in photographs of the process. As food systems are so complex, with a myriad of actors and underlying issues and outcomes, the T-Labs built on a systems approach that integrates thinking, reviewing and reflecting, and doing (see Drimie et al. 2018). A varied set of tools and participatory methods were carefully constructed by the facilitation team in order to create a space that could advance the T-labs objectives (see Table 3). This design was to allow concrete coalitions to form and ideas to be translated into action through building relationships and commitment for the actors to drive change. The two T-labs brought together a diverse group of actors that were actively engaged in creating alternatives in the food industry, such as indigenous and slow food activists, and informal food traders.

Invitations for the first T-lab were initially sent out over email to contacts from the Southern African Food Lab (SAFL) database, and further participants were invited using a snowball method. Before the first T-lab, a survey was sent out to the participants who had confirmed their attendance. The survey consisted of 5 open-ended questions focused on the activities that the actors are involved in within the food system, their expectations of the T-lab, and areas which they considered important intervention points. The intention was for this feedback to shape the T-lab process, however, due to timing of the feedback, it really only became possible to incorporate it into the design of the second T-lab.

Whereas the first T-Lab had been designed as a “safe-enough space” for participants with an interest or stake in the food system, the second T-lab was designed as a consolidation workshop and included both former and new participants. The first T-lab had resulted in some ideas and action pivoting on the intersections between niche, artisanal and fledgling projects intended to provide alternatives to the dominant food system. The consolidation workshop sought to build on and strengthen this. All participants from the first T-lab were invited, including new contacts in wider networks.

To address some of the challenges incurred during the first T-lab, facilitators involved participants in formulating the agenda, goals and objectives of the second T-lab right from the start. Instead of being a rigid structure or academic framework, it was an emergent process that was informed by the needs and interests of the participants. In turn, participants were more able to engage in discussions on how to find sustainable and practical solutions to the challenges they face within the food system, and seemed more comfortable with giving feedback on the process and making suggestions. As a part of this more flexible approach, the “ideas room” was set up as a physical space available to all participants at any time for deeper reflection and filled with coloured pens, wax crayons, clay, water, seeds and images from a food exhibition.

**Fig. 1:**
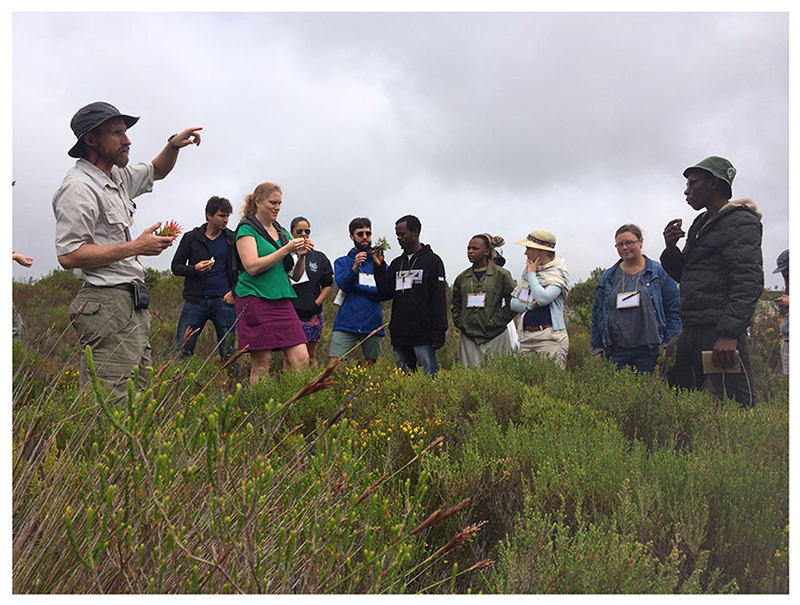
Participants on a learning journey through the fynbos at Grootbos Nature Reserve (Photo credit: Elke Markey)

A core aspect of the T-labs were the informal interaction sessions and a key component of their design was to ensure that important connections were made in the informal spaces, as well as during the more structured aspects of the lab. These are as important, if not more so, than the more formally structured programme of events. In both T-labs, all the participants were staying at the same self-catering venue and so cooked meals together. The focus of the first T-lab centred on indigenous foods, and by foraging for wild foods on their field trip (Fig 1.) and preparing these together, participants were able to engage with each other on a human level through the shared experience of ‘chopping and chatting’ (Fig. 3). As we had chefs in attendance, on the second evening we held a ‘cook off’ where chefs had different teams of sous-chefs to help them each to prepare a different dish. It was MasterChef, T-lab style, and we found it really helped to build connections.

**Fig. 2:**
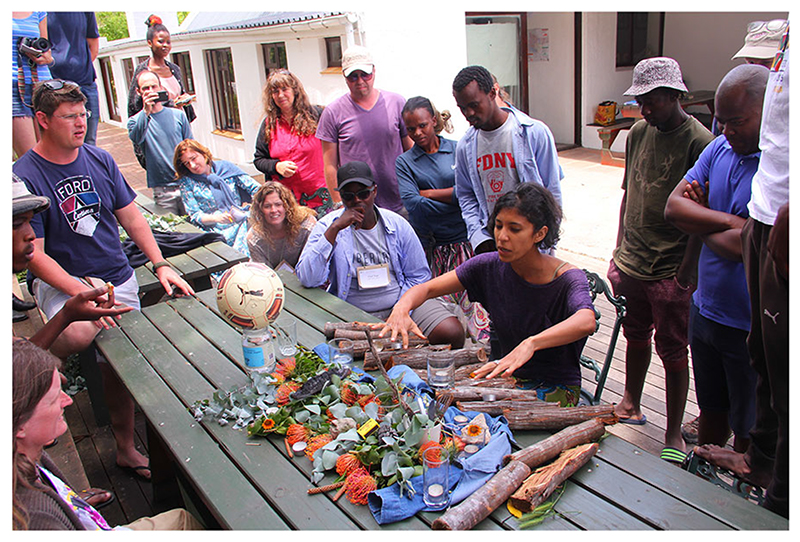
One group of participants explaining their vision of a better future food system using items they had collected around the venue (Photo credit: Megan Lindow)

**Fig. 3:**
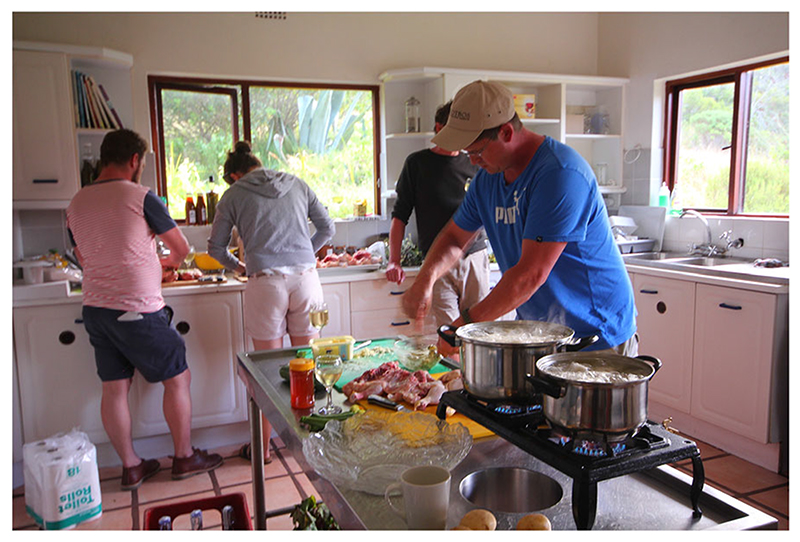
Participants in the kitchen ‘chopping and chatting’ (Photo credit: Megan Lindow)

Collective food preparation remained a big part of the second T-lab process, although there was no field trip for foraging local wild foods. Instead, it was done in a way that built an understanding of combining different foods, flavours and textures through experimentation and eating. There was a general sense that the critical social connections were made when everyone was tasked with collecting items from the environment for their group vision (Fig 2.), helping to prepare food (Fig. 3) or eating together. This was a theme throughout both T-labs described here and continued in the subsequent third T-lab held in May 2019 that focussed on coastal wild foods (Cramer et al. 2019). For more detailed information on the T-labs, please see Zgambo et al. (2018) and Zgambo et al. (2018).

### Follow-up interviews and observations

Soon after the first T-lab concluded, participants were asked to reflect on their experience and perception of the T-lab design in a post-workshop survey. This was especially important feedback for the facilitators to have going forwards as the process had been a first attempt at introducing the T-lab concept in a developing country context. It was also useful to reflect on whether the process achieved its purpose, the areas that needed improvement or maintenance, and who was missing from the room.

The post-workshop survey revealed mixed reviews of the T-labs from the participants. Positive feedback included that the T-lab had met participant expectations and was a great academic and practitioner collaboration. Some participants felt the T-lab was a much-needed intervention in the food system and was an eye-opening and motivating process. Many enjoyed learning about new and/or alternative food systems and were interested to incorporate more indigenous and local foods into their diet, grow medicinal plants, even incorporating a local menu at their restaurant and focusing on indigenous foods in their training programs. However, the survey also indicated a general dissatisfaction with the T-lab process, including its objectives, the way facilitators had handled the tensions in the room, the overly academic and lecture-like nature of the presentations and discussions, and lack of some key stakeholders in the room (i.e. government officials, especially since issues such as food policy were being discussed). These comments were noted and the design of the second T-lab was done in conjunction with a few key stakeholders from the previous lab and tried to address these concerns.

In a follow-up study, semi-structured, face-to-face interviews were conducted between April and July 2017 within the Stellenbosch and Cape Town area, each lasting on average between 45 min to an hour. Questions focused on collecting data that would help define an “alternative food system actor” within the local context, and to determine whether the T-lab process had been an effective tool of intervention in the local alternative food system as a means of tracking any emergent potential impacts. The following themes were discussed during the interviews:

1. The participants’ role within South Africa’s food system, what values define their work, and how explicit they are about these with their audience.
2. What they would like to achieve with their initiative/organization.
3. If they are addressing any sustainability or social injustice and inequality issues with their work, and how.
4. Involvement with their local communities.
5. If they found anything unusual about the T-lab process, and their willingness to participate in similar events in the future.
6. Ways in which they had collaborated with other T-lab participants after the process, or why not.
7. If the T-lab process has challenged their worldviews/values/beliefs about food, and how this might influence them and their way of doing.
8. If they would need to change something about themselves for them to contribute to the South African food transformation, and what they consider as potential hindrances to this transformation.

Although it would have been ideal to interview all participants from the T-lab, as the interviews were part of a Master’s thesis, time, spatial and financial restrictions were limited and so participants were chosen based on the roles in the alternative food system. For example, among the participants were four chefs, two retailers, a baker, three wild food innovators, four organic food farmers, two restaurateurs, a nutritionist, food activists, a food scientist, and researchers. Thus, for the interviews, one person in each role was randomly selected as a representative of that category. In addition, care was taken to have equal gender representation. However, due to unavailability of participants, only four of the ten interviewees were female.

In some ways, this categorisation was a limitation to the data collection process as serving similar roles in the food system does not imply that they share the same perspective. However, this method was effective in getting various opinions from across the food system, as well as varied feedback on potential T-lab impacts. These findings were triangulated with the survey responses and the informal conversations that were undertaken during the T-labs themselves in order to ensure as much relevant information was captured as possible.

Another, potentially more important limitation, is that this was a snapshot in time of the broader transformational process. As such, the information that was gathered and analysed could only refer to what happened directly after the T-labs and not how the broader impacts or transformative potential of the networks and coalitions- and their ability to influence planning-was enabled in the longer term. This is a major drawback in most projects looking at transformations, that by definition happen over time and not instantaneously (Pereira et al. 2020a). One way to overcome this limitation is to ensure that there is a continued relationship with the community that is formed through the T-lab process and to engage in ongoing processes with measurements of impacts over time. In the case of the Western Cape, this has been done with a third T-lab undertaken in May 2019 that shows a maturity both of the network, but also of the facilitators as we have ‘learned by doing’ over the past years of this research engagement (Cramer et al. 2019). The analysis of this, however, is not in the scope of this paper, but serves to illustrate how further engagement over a period of time can help to address some of the narrow analysis of a particular moment in time.

## Discussion of T-lab outcomes and learnings

The literature highlights several key aspects that enable transformation including:

1. Changing core state variables and key cycles of a system (Olsson et al. 2006)
2. Multilevel and multiphase processes of action such as a combination of activities and entrepreneurial innovations that can create new, more sustainable trajectories (Geels 2002; Westley et al. 2013)
3. Radical and continuous political re-alignments, social and technological innovations to build human capacity for the new system (Walker et al. 2004; Leach et al. 2010; Stirling 2014)
4. Repeated, collective participatory engagement, policy and institutional rearrangements (Olsson and Galaz 2012; Leach et al. 2013; Westley et al. 2013)
5. A window of opportunity, i.e. political will or widening cracks and tensions in a dominant system that can allow emerging innovations to permeate through (Olsson et al. 2004, 2006)

It is important to keep these characteristics in mind when designing and analysing processes for enabling co-production of knowledge and action for transformations – i.e., transformative spaces, using approaches such as T-labs (Pereira et al. 2018b; Ely et al. 2020). In the case of the Food System T-labs employed in the Western Cape, the aim of the labs was to foster networks of engaged individuals who were innovating in isolation (3) and to take advantage of the Western Cape government’s food and nutrition strategy as a window of opportunity to get political buy-in (5). It was recognised that there was a need for an ongoing process, and that there would have to be at least two labs, if not more, if new systemic configurations were to emerge (4). Finally, the labs were explicit in not wanting to bring too many levels of engagement into the space-it was considered that there was sufficient diversity in the alternative food space at the local level that bringing in more powerful players was not thought to be strategic-therefore the process did not explicitly engage with points 1 and 2, but acknowledged these dynamics in the design of T-labs by ‘Seeing the System’.

In this section, we explore the findings from the perspective of learning about the design of a T-lab as a transformative space that can build new relationships and strengthen the networks within the alternative food system. We focus mainly on the methodological learnings from these events, but also situate the findings within the broader lab literature. Another point of interest was to see how the outcomes from the T-lab could feed into the ongoing provincial planning process that was seeking to incorporate novel methods for including diverse stakeholder voices in its policy development process. In section 5.1.2., we describe a specific outcome from the T-labs, a Food Charter, that fed directly into the provincial planning process.

### Reflections on the T-lab design: the importance of diverse perspectives

**Fig. 4:**
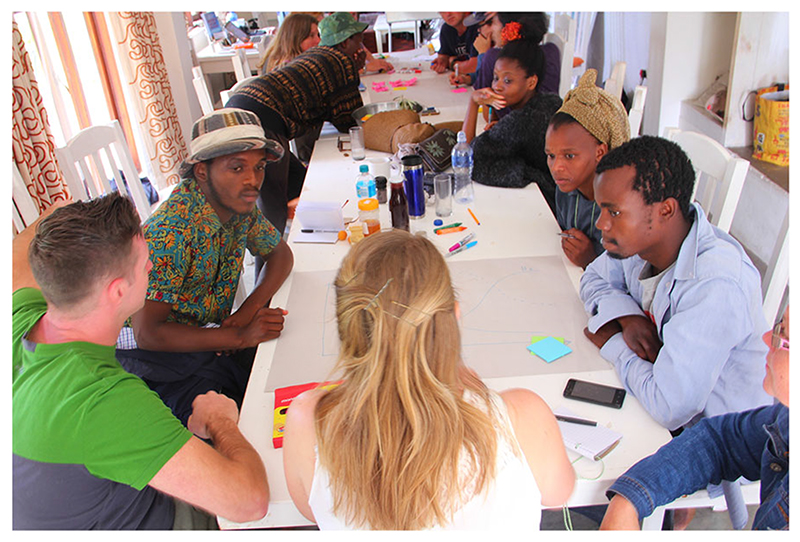
One of the more structured sessions where groups of participants recorded their often heated discussions on posters with sticky notes (Photo credit: Megan Lindow)

Bringing together a diverse grouping of people within the same system helps bring many perspectives into the room, which is useful for addressing complex challenges (Westley et al. 2013; Biggs et al. 2015). However, with diversity comes different opinions and worldviews, even among people within the same sector and background. Care must be taken to navigate and address these differences accordingly and not let them stifle the (T-)lab process (Westley 2013; Ely and Marin 2016). As facilitators we did not feel in advance that the process was too highly structured. But feedback from participants during the first T-lab had us constantly restructuring sessions so as to try and give participants some freedom, i.e. the concept of “fostering reflexivity” (Pereira et al. 2015). There were concerns raised about the T-lab being a ‘Western concept’ and that the facilitators were unaware of the nuances of the South African food system. This was partly a reaction to a facilitation team, which had not previously worked together, but it was also a result of the experimental process that was used (Fig. 4). We needed to learn at an exponential rate about how to hold the space to allow for an emergent outcome that was driven by the participants themselves – but that at least would yield some sort of outcome. Whilst it is difficult for an open process to ‘fail,’ it was often at the forefront of the facilitators’ minds as issues around race, power dynamics, cultural backgrounds and trust raised their heads at different points. These concerns are all central to a process that seeks to create a momentum amongst a diverse group of people and comes to the fore rapidly in a country like South Africa where discrimination and marginalisation is such a fundamental aspect of the country’s history.

The reflections from the second T-lab were generally more positive. By listening to feedback, actively responding and being open about the process, it seems as though we were able to create a space for true engagement and trust-building, (Olsson et al. 2014, 2017):

*“My experience was good. I think this workshop was powerful. Compared to other workshops which were structured from the top to the bottom. But this time we could feel that things were starting at the roots.”*

According to the participants, the bottom-up approach allowed them to open up and think:

*“This space is necessary and effective in its laid-back nature as it allows people to open up and think slowly. It was both fluid and productive.”*

A theme that emerged from the reflections on the second T-lab was the opportunity to share diverse opinions. According to the participants, the diversity of their areas of expertise and race was important for the realization of what can be done differently in the food system for different population groups. For some of the participants, the diversity provided answers to questions, a space to influence the influencers and to encourage active citizenship. These findings talk directly to the ‘enhanced capacities such as knowledge gain’ output of transdisciplinary sustainability research in the framework by Wiek et al. (2014). As one workshop participant put it:

*“It was my first experience at a T-lab. It was useful and constructive: the networks and collaborations especially. I got diverse opinions and the missing links were filled.”*

*“What we are dealing with is far more complex than I thought it was.”*

Such a finding reinforces that building social capital is an important aspect of the lab process (Schäpke et al. 2017). It also underscores the findings of how creating and maintaining spaces for learning as a core role for researchers operating in this complex action research processes (Wittmayer and Schäpke 2014).

Ensuring representativeness is crucial when a process aims to influence government decision-making (Lipmanowicz and McCandless 2014; Ziervogel et al. 2016). Who to invite remains a tricky question and should be dictated by the planning question being deliberated and the level of vulnerability that some actors might have in the presence of more powerful actors (Pereira et al. 2020a). If incorporated as key strategies to help policy and planning to be more inclusive (Hebinck et al. 2018), the legitimacy of T-labs will come under scrutiny and the justification for their construction, who convenes them and who is invited in to participate, must be transparent and designed to meet needs in an open and democratic fashion. A key challenge is to open up a space for productive collaboration and interaction between diverse actors with the intention that there may be actionable outcomes with which decision-making actors can engage (Tengö et al. 2017; Hebinck and Page 2017). It is, however, sometimes more effective to have fewer people with less overt differences in order to be able to reach some kind of actionable plan. The question of how many levels of representation (i.e. national, provincial, community) are necessary for an effective lab will be dictated by the question. In this case, the focus was on alternative urban food system. It was intended to be disruptive, but as there was already a lot of diversity in involving just community level actors, no higher system level actors were invited. However, for future iterations-now that there is an established network - other actors from different parts of the system can also be invited to take part.

As T-labs are not static ‘events’, but processes to catalyse ongoing change, it is also important to note that not all aspects of transformation will necessarily be dealt with through one process (Pereira et al. 2020a). As indicated above, there was a feeling amongst the group that at some point it will be necessary to bring other actors into the space in order to interact with the dominant regime. Parallel processes of transformative interventions (not in the form of T-labs, but as dialogues) have been undertaken in the South Africa food system (Drimie et al. 2018). By aligning these processes, it is possible for some to deal with certain characteristics of transformation whilst others are concerned with different challenges. What is critical is to ensure that the processes align and are not undertaken in an ad hoc fashion. However, given the funding constraints for such processes, it can be difficult to ensure a long-term strategic plan and this is one of the weaknesses of such approaches (see the contraints discussed in Pereira et al. 2020a).

### Reflections on the T-lab design: connecting new partnerships and collaborations to everyday actions

In the overall research process, it was envisaged that the process would build new relationships and strengthen the networks within the alternative food system (Tengö et al. 2017; Hebinck et al. 2018; Marshall et al. 2018). It would thus serve as a platform for enabling solutions to the challenges faced by the participants that could feed into the provincial government’s strategic focus on food and nutrition security. Despite the contestations that took place during the first T-lab, new relationships were also being formed and networks were being built. There are demonstrable examples of this both articulated by participants, but also showcased through subsequent collaborations. During the work, the participants formed relationships. One participant had this to say:

*“I was encouraged that I am not alone – there are a lot more people longing for the food revolution to take place. At times, I am tempted to quit and do something else that will make me more money. (But) they motivated me to continue farming to do something about the country/environment*.

*After (the T-lab), I started working with an indigenous food innovator and activist on an indigenous garden at the Institute, and with some urban farmers to erect community gardens in a nearby community centre.”*

From these relations, participants planned to collaborate on their work. For example, the chefs from a well-known restaurant planned to work together with the local food innovator to incorporate local menu at their hotel’s restaurant. An artisanal baker promised to build an oven at a community garden in an informal settlement. The latter has been a huge success in growing the community garden as a space for innovation and education about agro-ecological farming, nutrition and the importance of healthy diets in the community.

The respondents also realised the importance of sharing. They recognised that they have something valuable to share from their personal experience and what they had learned at the workshop:

*“Share what I have learned with as many people as possible”*.

The T-Lab was also beneficial for networking people in the alternative food system. The participants noticed that each of them was involved in the food system, but in their silos. The T-lab provided a bridge to connect these alternative food vendors. As this participant remarks:

*“It was great to meet with people from the same industry and with the same stories.”*

The participants also mentioned that the T-Lab gave them encouragement and recognition. The creation of a common group of alternative food vendors enhanced the participant’s sense that they are not alone.

*“The T-Lab has given us a recognition that we don’t receive from others because we are challenging them on fundamental issues that they are unlikely to embrace. I think this recognition is great.”*

Although these reflections provide evidence of new relationships and collaborations being forged, it is difficult to tell with follow-up interviews so close after the labs themselves, what sort of impact these spaces really had on the food system in Cape Town. After the T-lab, participants tend to go back to their day-to-day lives, unless they have certain incentives to connect with others or follow up on their collaboration plans. This suggests a disconnect between the T-lab space and the “outside world”, especially if trust or a sense of leadership has not developed to motivate them forward without being prompted to do so (Olsson et al. 2006; Olsson 2017). As a result, many of the ideas, initiatives or ways of thinking or doing that emerged during the first T-lab process, remain unexplored.

It is essential that facilitators endeavour to conduct T-labs in a manner that is as close to real-life situation of participants as possible (Bergvall-Kåreborn and Stahlbrost 2009; Bergvall-Kåreborn et al. 2009). This can enable participants to easily transition or implement ideas and innovations from the T-lab into their everyday life and work. T-labs have the potential to set things in motion, i.e. prepare for change in a transformation process, and with widening tensions in the dominant food regime, windows of opportunity are opening for an accumulation of such niche activities to influence the regime in a way that has not been possible before. However, ensuring that the solutions, networks and ideas continue to bear fruit beyond the span of the lab process remains an ongoing challenge, especially when resources are scarce. Otherwise, the ideas, initiatives or “ways of thinking or doing that exist, at least in prototype form” (Bennett et al. 2016: 442) that emerge during the (T-)lab process remain unexplored.

It was generally seen to be more effective when the facilitators allowed the T-lab to be an emergent process informed by the needs and interests of the participants, i.e. getting them involved in formulating the agenda, goals and objectives of the T-lab. Instead of being a rigid structure or academic framework, participants are more likely to participate and contribute to the process when they are involved in it from the beginning. They may also be more apt to finding sustainable and practical solutions to the challenges they face. While T-labs need further development to reach their potential to “juxtapos(e) the old and the new, the technological and the social, and the political and the economic”(Westley 2013: 6), they can be suitable spaces for building trust and comradery, strengthening existing structures within a system, and as a platform for collaboration.

### The T-labs and the broader planning process

In 2016, the Western Cape government’s Food and Nutrition Security Strategy acknowledged the need for a set of principles to guide their strategy (Western Cape Government 2016). Unfortunately, explicit mention of a Food Charter was omitted from the final strategy. It was understood that the provincial cabinet rejected the idea of a Food Charter, which had been included in an earlier draft of the Strategic Framework, as it was deemed ‘not practical enough’ to underpin the Strategy’s ‘legacy phase’. The province wanted to be seen as pursuing action and not principles.

During the second T-Lab, the group of “alternative food system” actors and activists again raised the issue of a Food Charter. This had previously been suggested by activists and academics working in this area, but had not been able to garner much momentum. The T-lab participants suggested that building a legitimate Food Charter from the ground up would provide a powerful way to guide future engagement with policy processes as well as to guide the conceptualisation and implementation of practical action on the ground. It was argued that this Food Charter should be based on the South African constitution and address issues around land. The charter would be a means to hold government and other actors to account and would not be owned by any organization. As a result of these discussions at the T-lab and having some of the right people at the right place at the right time, it was possible to operationalize the Food Charter in a way that had not yet been undertaken by individual societal actors.

Seven participants of the T-Lab committed themselves to helping develop the Charter, although funding and facilitation were identified as a constraint. In response, the facilitation team approached the Centre of Excellence (CoE) in Food Security based at the University of Western Cape (UWC) to consider funding a project on a Food Charter. The premise was that a Charter was a potentially important way to build awareness about the food system and consider new guidelines for its governance through establishing a set of principles and objectives on which decisions about food could be based. The CoE agreed to provide funding for a scoping study that would include a careful process of stakeholder engagement in order to ensure its legitimacy. The engagement process was subsequently run by the Southern Africa Food Lab drawing in one of the participants of the T-Lab to undertake the work. The intention was that the scoping study would lead to a working group being established to take this into provincial and other forums. The work was supported by a postgraduate student based at the University of the Western Cape.

Discussions around a Food Charter highlighted the need to generate more explicit connections between bottom up, co-produced processes and more formal governance structures. In essence, it was argued by participants of the T-lab that a Food Charter would ensure that the inclusion of grassroots organisations in discussions and decisions about the Cape Town food system. The deep thinking that goes into the creation of a T-lab as a transformative space through which potentially transformative systemic interventions can emerge is one way of initiating transformative change (Pereira et al. 2018b). It also highlights how carefully curated processes such as a T-lab can generate ideas and commitment for new responses to the food system, such as a Food Charter, which is often in contrast to what government institutions can do to generate similar types of interventions. By creating an enabling space through which the outcomes of a T-lab can be fostered as experiments, and by opening up places for interaction between these co-produced, diverse spaces and more formal, government institutions, there is potential for guiding transformative change onto a more sustainable and desirable trajectory.

The scoping study for the Food Charter was completed at the end of 2019 and is in process of being published by the Centre of Excellence in Food Security at the University of Western Cape. It fed directly into the Food Declaration on a Good Food Future developed as part of the Western Cape Food Governance Community of Practice. The Declaration identifies key values and principles that might guide a broad coalition of actors in building a new food system in the province. The Food Charter research looked critically at the history of charters and similar instruments in South Africa with a view to considering how viable it would be to develop a Food Charter in the Western Cape. The research raised concern about the legitimacy of a Food Charter, advising for a shift of focus onto a Declaration to stimulate debate with wider actors. This process continues to highlight how initiatives stemming from a T-lab process that is fundamentally about the co-production of knowledge can reignite the impetus for more engaged participation in planning processes.

## Concluding thoughts: reflections on the implications of transformative spaces for urban planning

The need to include food in urban planning agendas is becoming increasingly recognized (Cabannes and Marocchino 2018). Although until recently, urban planners emphasised the usual urban priorities like public transportation and decent housing, since the new millennium they have started to pay more attention to food, which, ironically, was the magnet for creative city planning just a couple of centuries ago (Cabannes and Marocchino 2018). Cape Town is a signatory to the Milan Urban Food Policy Pact that commits city governments to develop sustainable food systems that are inclusive, resilient, safe and diverse.^1^ Food is therefore firmly on the urban planning agenda, however, as with all systemic challenges, how to effectively integrate multiple actors and sectors across different scales is not easily addressed. Ensuring effective participation of all stakeholders and giving all citizens a voice in how their food system is managed is one important step in the process (Bellwood-Howard et al. 2018; Song and Taylor 2018).

The overarching question for this study was how a T-lab design can help to create a space to enable food system transformation in the South African context. The T-labs, as participatory processes, enabled a number of change-agents drawn from a diverse group of stakeholders to be brought together in a creative, generative process to develop outcomes with which policy makers could shape interventions. The food system was used as an entry point to bring diverse stakeholders together to learn about the complexity of the Cape Town food system, to consider ideas for responding, and to see how governance can support action that might lead to positive transformative change within an urban environment.

A key outcome of the process was the deepened understanding amongst these actors of the broader food system. The inter-connections between the participants reinforced the importance of the cross-pollination of ideas and understanding that the process facilitated. This emerged particularly when the participants considered what an “innovation” or response to their situation entailed, as opportunities through the linkages and alignment between participants became more explicit. This reinforced a key learning around the importance of explicitly building networks and seeking connections and the alignment of resources.

We found one of the key tensions was needing to plan and manage for specific outputs in the short-term, especially in government and business environments, versus the need to create enabling conditions that allow for the emergence of new and more sustainable and equitable system states over an undetermined time period. Another challenge was how to foster diversity and difference in opinions whilst simultaneously acting for ‘the common good’ as well as to seek ways to scale impact appropriately across different contexts (Ely et al. 2020). The inclusion of certain actors, the sectors that they represent and the power dynamics that they bring into the room are very important considerations - as with all participatory processes - but especially when there is a direct link to governmental planning initiatives (Darrah et al. 2018; Dinesh et al. 2018; Mangnus et al. 2019).

Applying the T-lab process within the unique political and historical context of post-Apartheid South Africa provides insight into how learnings from diverse contexts in the global South can help open up new knowledge for addressing sustainability transformations globally. The emphasis on trust-building despite highly distorted food systems in southern Africa forms a large part of collaborative, co-production processes in the region (Drimie et al. 2018; Mukute et al. 2018). Addressing historical concerns and power dynamics in real-time in these lab environments requires artful facilitation to be able to make these dynamics explicit without derailing the process (Drimie et al. 2018). At the same time, the convening researchers are themselves constantly learning and having to adapt to changing circumstances, which means that these kinds of transformative space-making processes are rarely without flaws (Pereira et al. 2020a). However, documenting and learning from mistakes and being open to reflection and alternative ways of doing things are vital for improving research in this space going forward (Ely et al. 2020). This is one of the main objectives of writing up this paper, so that the learning that was generated from the T-lab process can be shared with those who may be wanting to engage in similar processes.

The food system T-labs provided an entry point that opened up much of the complexities of planning for transformative change in urban environments, but also highlighted some of the potential solutions for navigating urban sustainability transformations more generally. In the future, T-labs could be institutionalized to complement standard planning processes. When there is a particularly complex question that requires participatory deliberation with a set of change-makers who understand the field, T-labs could be designed to provide innovative insights from stakeholders as to how to proceed from a governance perspective. As these interventions and networks move forward, it will be important to follow how much the T-lab outcomes are able to inform provincial planning processes. If so, how such changes in urban planning processes are able to filter up to national planning processes so that potentially transformative solutions are not prematurely cut-off by constraining national strategies, is important for future research.

Finally, it is important to emphasise that as the diversity of documented case studies applying T-lab processes grows, so will the richness of our knowledge and insights on how to enable sustainability transformations. This T-lab process has showcased how innovative, participatory processes that are action-oriented as well as policy-relevant can be facilitated-provided that there is buy-in from the community. However, it also highlighted many tensions when operating in spaces of extreme inequality and historical dispossession. Experimenting with more such processes, particularly in underserved areas of the world, will ensure wide learning. Sharing these experiences will add to the growing body of knowledge on how to enable urban transformations towards a more sustainable and equitable planet.

## Figures and Tables

**Table 1:** T-lab 1

The first T-lab was conducted at Grootbos Nature Reserve, 150 km outside Cape Town, from 27 to 30 November 2016. The T-lab was designed as a multi-actor innovation process with the goal of better understanding pressing issues in the local food system, building coalitions of change, generating ideas and commitment, and testing these ideas on the ground. There were 35 participants in total; including chefs, researchers, artists, food activists, producers, retailers, food innovators, an anthropologist, food scientist and an artisanal baker. Four researchers from the Centre for Complex Systems in Transition (CST) at Stellenbosch University, the Southern African Food Lab (SAFL), and Stockholm Resilience Centre (SRC) facilitated the T-lab. The T-lab had three phases 1- Seeing the system: which included a learning journey *en route* to the venue and a guided tour of the areas foraging for edibles in the Fynbos landscape and learning about the indigenous flora (Fig. 1). 2- Visioning a better future: which involved participants foraging for things (e.g. kitchen utensils, cutlery, stones, twigs, leaves and fruit) to create a vision of their desired food future through the creation of an “artefact”. The Three Horizons framework was also employed as a device to see how we could get from present roles and routines to a more transformed system (Fig. 2). 3- Committing to actions: which involved the participants offering what they were able to do differently to spur the change they wanted to see and to forge networks and relationships with some of those in the room and beyond in order to effect change. Challenges included a sense of uncertainty from the participants on some aspects of the T-lab process, and how it had been conducted. Other concerns were that the objective of the event had not been clearly stipulated, and the language used during presentations was overly theoretical in nature.

**Table 2:** T-lab 2

The second T-lab took place from 19 to 21 July 2017, at Nine Oaks, Paarl, a venue 70 Km from Cape Town. The goal of the 2nd T-Lab was to further develop and strengthen the trust between participants in the emerging coalition of change that would then enable them to continue to define and implement breakthrough solutions. 22 participants attended the second T-lab, with 7 coming from those that had attended the first T-lab. Participants included: permaculture specialists, food and land activists, restaurateurs, urban farmers, and a representative from the informal traders’ association, researchers, anthropologist, and indigenous food innovator. Only two of the four researchers from the first T-lab (from the CST and the SAFL) facilitated the process. The Consolidation Workshop was based on three distinct movements that unfolded over 2 days. These were: Sensing the system Letting Go (old ways of working) Letting Come (emerging innovation) Having learned from the experience of the previous T-lab, this lab was designed with much more humble ambitions and as such did not experience as many challenges as the first one had. There was an overall positive response from participants when invitations were sent out. This allowed for a subsequent T-lab to take place on 2–3 May 2019 in Cape Town with a further subset of participants and new invitees (See Cramer et al. 2019).

**Table 3:** Key activities during the T-lab processes conducted in the Western Cape

Activity	Objective
Learning journeys	These are important tools in developing the “collective leadership capacity [that draws] together all key stakeholders and involve [s] them in a process that begins with uncovering common intention and ends with collectively creating profound innovation on the scale of the whole system” (Scharmer 2010). Here, they were used to help participants to identify images that epitomise the challenges of the current food system in the landscape between their departure point and T-lab venue, and why.
Foraging and a guided tour of the surrounding area	To (re) connect people with the local nature, and to learn about the different wild foods in the area. This was also a good way to get people thinking about some of the resources they may have at their disposal in their own localities.
Provocation with realities of the dominant food system	To help participants determine what is wrong with the current/dominant food system, and what about this system can be connected to the alternative food system.
Three Horizons framework	This is a heuristic that can help participants to think about transformative pathways towards more desirable futures and how this future is linked to the present (Sharpe et al. 2016). It was used to illustrate how change can be projected from what is to what could be within the food system. It also informed the visioning exercise on alternative food system futures that participants would like to see.
Visioning exercise	Participants were divided into groups of six or more to creatively illustrate (using kitchen utensils, cutlery, stones, twigs, leaves and fruit) how they envision future food systems, and what impact their innovation could potentially have (Fig. 2).
Group activities i.e. cooking together, chef cook-off	Although not mandatory, group activities such as cooking together were a large part of the T-lab process. Participants were involved in the cooking, cleaning or setting up of tables at all mealtimes. The role of indigenous foods in addressing hunger, food insecurity and nutrition challenges in the Western Cape (and at national level) were highlighted throughout the T-lab process. There was also a cook-off that was a competitive yet playful means of participant interaction (with food and each other) (Fig. 3).

## Data Availability

The thesis on which this paper is based is available here: http://scholar.sun.ac.za/handle/10019.1/103445

## Science Highlights

T-Labs are specifically designed to guide transformations in social-ecological systems towards sustainability.

T-labs are messy, emergent processes and to be most effective they should be informed by the needs of the participants.

As transformative spaces, T-labs are ongoing processes of engagement with changemakers, not single events.

### Policy and Practice Recommendations

T-labs can enable inclusive planning by enabling innovative, co-produced knowledge to feed into policy.

The legitimacy of T-labs requires a transparent justification for their design, convening and who participates.

T-labs can complement standard planning processes when there is a complex issue that needs participatory deliberation.

## Footnotes

1
http://www.milanurbanfoodpolicypact.org/

## Abbreviations

SES: Social-Ecological Systems
T-lab: Transformation Lab
